# Our Experience With Left Colon and Rectal Cancer Surgery and the Impact of Preoperative Sarcopenia on Complication Rates

**DOI:** 10.7759/cureus.45209

**Published:** 2023-09-14

**Authors:** Huseyin K Rasa, Ayhan Erdemir

**Affiliations:** 1 General Surgery, Anadolu Medical Center Hospital, Kocaeli, TUR

**Keywords:** surgical complications, patient outcomes, rectum cancer, left colon cancer, sarcopenia

## Abstract

Background: Evidence about the importance of sarcopenia in patients operated on for gastrointestinal cancers and that it may have both early and long-term impacts is expanding. In our study, we aimed to evaluate the impact of sarcopenia on the outcomes of the patients we operated on for left colon and rectum cancer.

Methods: We retrospectively evaluated the electronic records of 38 patients operated on for left colon and rectal cancer between 2010 and 2020, and demographic variables, clinical stages, laboratory tests, body mass index (BMI), psoas muscle index (PMI), pathological stages, and Dindo Clavien complication scores were interpreted. We also assigned our patients into two groups according to their preoperative PMI values. We compared the first group of 12 patients with preoperative sarcopenia with the second group of 26 patients without preoperative sarcopenia.

Results: Of the 38 patients who underwent curative surgery for left colon and rectal cancer, 20 were female and 18 were male. The median age of the group was 59.9 years. The most common tumour localization was in the rectosigmoid region in 17 patients, and the tumour in 6 patients was in the left colon. Therapy had been initiated with neoadjuvant treatment in 19 patients. At the preoperative evaluation, sarcopenia was present in 12 patients. Thirty-four patients underwent robot-assisted surgery. Postoperative pathologies were reported as stage 3 in 15 patients. Complications were reported in 17 patients, and nine were minor (Dindo-Clavien score < 3), but in eight patients, they were moderate to severe (Dindo-Clavien score ≥ 3).

When the first group, 12 patients with preoperative sarcopenia, and the second group, 26 patients without preoperative sarcopenia, were compared, the patients with sarcopenia were found to be older (p=0.001), and male patients were in the majority (p=0.017). The postoperative follow-up of 12 patients with preoperative sarcopenia revealed that 7 (58.8%) had complications. Complications were observed in 10 (38.4%) patients in the second group. When the two groups were compared, the risk of developing complications was significantly higher in the sarcopenia group (p=0.016). Only one patient in the first group had moderate to severe complications, but seven patients without sarcopenia had moderate to severe complications.

Conclusion: Our study revealed that many patients we have operated on for left colon and rectal cancer have preoperative sarcopenia for which we should care. The sarcopenia rate was higher in males and elderly patients, and the risk of overall postoperative complications increased significantly in patients with preoperative sarcopenia. In consequence, the results of our study provide evidence that preoperative sarcopenia status is an important parameter to determine the risk status of the patient, and patients with preoperative sarcopenia should be monitored more closely. Thus, we may be able to diagnose and intervene early in the complications.

## Introduction

The nutritional status of the patients has a decisive effect on the postoperative complication rates. There is growing evidence that nutritional parameters have predictive value for patient outcomes and that sarcopenia is a critical determinant [1]. In patients who underwent surgery for colorectal cancer, sarcopenia increased the risk of postoperative complications and negatively impacted survival [2]. Evaluation of sarcopenia in the perioperative period and closer monitoring of the patients who are at risk will enable early detection of complications, especially anastomotic leaks [3].

Our study evaluated 38 patients diagnosed with and operated on for left colon and rectum cancer between 2010 and 2020. We aimed to define the demographic variables correlated with preoperative sarcopenia and evaluate the impact of sarcopenia on patient outcomes.

## Materials and methods

All patients diagnosed with left colon and rectum cancer in our hospital are evaluated by the 'Gastrointestinal Tumor Board' before treatment. The clinical status of the patients, colonoscopic findings, and pathologies are discussed, and according to MRI and PET-CT findings, we decide the stage and tailor their treatment plan accordingly. In these weekly meetings, the treatment responses of the patients who have decided to initiate treatment with neoadjuvant chemoradiotherapy are also discussed, and the timing of their surgery is planned.

In our study, the electronic records of 38 patients who were operated on consecutively between 2010 and 2020 in our hospital for left colon and rectum cancer were evaluated retrospectively. Demographic characteristics of the patients, the extent of their disease, laboratory tests, neoadjuvant chemotherapy and radiotherapy information, body mass index (BMI), psoas muscle index (PMI), pathological stages, and Dindo-Clavien complication scores were evaluated.

PMI was calculated by the ratio of the mean of both psoas muscles in cm^2^ to the patient's body surface area in m2 in the third lumbar vertebra localization, which corresponds to the umbilical fossa, in the preoperative computed tomography examination (cm^2^/m^2^). Since this rate is different between men and women, considering the study of Hamaguci, sarcopenia was defined as less than 6.36 cm^2^/m^2^ in men and below 3.92 cm^2^/m^2^ in women [4].

We assigned our patients into two groups according to their preoperative PMI values. We aimed to understand the impact of sarcopenia on variables and complication rates by comparing the first group of 12 patients with preoperative sarcopenia and the second group of 26 patients without preoperative sarcopenia.

The study was conducted following the Declaration of Helsinki (revised in 2013), and the Anadolu Medical Center Review Board and Ethics Committee waived the need for ethics approval for studies based on retrospective data or file analysis; hence, individual consent for this retrospective data analysis was waived.

In the descriptive statistics of the data, mean, standard deviation, median, minimum, maximum, frequency, and ratio values were used. The distribution of variables was measured with the Kolmogorov-Kimirnov test. Independent sample t-tests and Mann-Whitney U-tests were used to analyze quantitative independent data. The chi-square test was used to analyze qualitative independent data, and the Fischer test was used when the chi-square test conditions were not met. The SPSS 28.0 program (IBM Corp., Armonk, NY) was used in the analysis.

## Results

Of the 38 patients who underwent curative surgery for left colon and rectal cancer, 20 were female and 18 were male. The median age of the group was 59.9 years. The most common tumor localization was in the rectosigmoid region in 17 patients (44.7%), and the tumor in 6 patients (15.8%) was in the left colon. Neoadjuvant treatment was applied to 19 patients (50%). At the preoperative evaluation, sarcopenia was present in 12 patients (31.5%). Our preference for the surgical treatment of colorectal cancer patients is minimally invasive, and by this, in 34 patients (89.5%), robot-assisted surgery and in 1 patient (2.6%), laparoscopic surgery had been performed. When the postoperative pathologies were evaluated, it was seen that 15 patients (39.5%) were reported as stage 3 (3 patients 3a, seven patients 3b, and five patients 3c), and 12 patients (31.6%) were reported as stage 1. Complications were reported in 17 (44.7%). Nine (23.6%) were minor (Dindo-Clavien score < 3), but in 8 patients (21%), moderate to severe (Dindo-Clavien score ≥ 3) (Table 1).

**Table 1:** Demographics of 38 patients operated for left colon and rectal cancer.

	N (%)
Median age (years)	59.9
Gender	
Female	20 (52.6%)
Male	18 (47.3%)
Localization of the tumor	
Left colon	6 (15.8%)
Rectosigmoid	17 (44.7%)
Mid-rectum	8 (21%)
Distal rectum	7 (18.4%)
Operation type	
Robotic surgery	33 (86.8%)
Laparoscopic surgery	2 (5.3%)
Open surgery	3 (7.9%)
Pathologic stage	
1	12 (31.6%)
2	7 (18.4%)
3	15 (39.5)
4	4 (10.5%)
Patients with neoadjuvant therapy	17 (44.7%)
Preoperative sarcopenia status	
Present	12 (31.5%)
Absent	26 (68.4%)
Patients with complications	17 (44.7%)
Minor (Dindo-Clavien <3)	9 (23,6%)
Moderete-severe (Dindo-Clavien ≥3)	8 (21%)

When the first group, 12 patients with preoperative sarcopenia, and the second group, 26 patients without preoperative sarcopenia, were compared, the patients with sarcopenia were found to be older (p=0.001), and male patients were in the majority (p=0.017) in the sarcopenia group. The tumor localization of the patients in both groups was similar (p=0.383). There was no significant difference in clinical stage (p=0.396) and other laboratory tests, mainly serum albumin levels (p=0.565). There was no difference between the preoperative BMI values of the groups (p=0.293), but by the methodology, the preoperative PMI values of both groups were found to be significantly different (p=0.002) (Table 2).

**Table 2:** Comparison of variables between the patients with preoperative sarcopenia with the second group, patients without preoperative sarcopenia. t: t-test; x^2^: chi-square test.

	Patients with preoperative sarcopenia (n:12)	Patients without preoperative sarcopenia (n:26)	p-value
Median age (years)	63.3	58.4	0.001^t^
Gender			0.017^x2^
Female	2	16	
Male	10	10	
Localization of the tumor			0.383^x2^
Left colon	2	4	
Rectosigmoid	6	11	
Mid-rectum	2	6	
Distal rectum	2	5	
Surgery			0.367^x2^
Robot-assisted	11	23	
Laparoscopic	0	1	
Open	1	2	
Neoadjuvant therapy	10	9	0.161^x2^
Female	10	4	
Male	0	5	
Pre-neoadjuvant therapy BMI (kg/m2)	30.9	26.68	0298^t^
Pre-neoadjuvant therapy PMI (cm^2^/m^2^)	6.0	6.83	0161^t^
Preoperative BMI (kg/m^2^)	27.4	27.0	0.293^t^
Preoperative PMI (cm^2^/m^2^)	4.67	6.51	0.002^t^
Female	3.25	5.29	
Male	4.95	7.55	
Number of patients with complications (%)	7 (58.8 %)	10 (38.4%)	0.016^x2^
Minor (Dindo-Clavien < 3)	6	3	
Moderate to severe (Dindo-Clavien ≥ 3)	1	7	

Ten of the twelve patients with preoperative sarcopenia received neoadjuvant treatment; this number was 9 in 26 patients without preoperative sarcopenia (p=0.161). When the subgroups of patients receiving neoadjuvant therapy were compared, no difference was found between the pretreatment BMI values of the two groups (p=0.298). There was a decrease in BMI values in both groups during the treatment process. However, when the preoperative BMI values of these subgroups of patients receiving neoadjuvant therapy were compared, no difference was found between the groups (p=0.293) (Table 2).

We had 12 patients in the preoperative sarcopenia group, and it is seen that the most frequently performed operation in this group of patients was robotic low anterior resection (LAR) with eight patients. Three of those patients had an ileostomy due to the ultra-low anastomosis. Robotic hemicolectomy was performed in three patients and open hemicolectomy in one patient. Seven complications developed (58.8%), six mild, and one moderate to severe. One of the patients who developed mild complications was female, and five were male. The complications were postoperative fever, urinary retention, and atelectasis, and two patients had superficial surgical site infections. An organ/space surgical site infection developed in one male patient in whom we performed an open hemicolectomy, which was the group's only "moderate to severe" complication, and this "left hemicolectomy" patient had no neoadjuvant therapy.

We had 26 patients in the non-sarcopenia group. Robotic LAR was performed on 12 of those 26 patients. Since the anastomosis in seven patients was ultra-low, a protective ileostomy was also performed during the index surgery. Robotic abdominoperineal resection (APR) was performed in four patients; robotic hemicolectomy was performed in seven patients; and laparoscopic APR, laparoscopic hemicolectomy, and open hemicolectomy were performed in one patient each. A total of ten complications developed (38.4%). Of the three patients who developed mild complications, one was female and two were male. The problem in two patients was fever in the postoperative period, which resolved spontaneously. A surgical site infection developed in the perianal site in the patient who underwent laparoscopic APR after neoadjuvant chemoradiotherapy. The number of patients who developed mild to moderate complications was seven (27%), four females and three males, and six of those patients underwent neoadjuvant chemoradiotherapy. A patient who underwent robotic LAR without an ileostomy developed an anastomotic leak and was re-operated for source control. Abdominal lavage and an ileostomy were performed. In a patient who underwent robotic LAR, an intra-abdominal abscess developed and was drained by interventional radiology. Pneumonia in one patient who underwent robotic APR was successfully treated. A deep surgical site infection in the perineal area developed in one patient who underwent robotic APR. One patient encountered complications related to the protective ileostomy, while another patient's borderline renal functions deteriorated in the postoperative period, requiring treatment to be extended due to renal insufficiency. We had "postoperative sepsis" in one patient but could not define the etiology, and it improved with appropriate empirical medical treatment. When the two groups were compared, the risk of developing complications was significantly higher in the first group (p=0.016). Contrary to what was predicted, only one patient in the first group had moderate to severe complications (Dindo-Clavien ≥ 3). In contrast, seven patients in the group without sarcopenia had moderate to severe complications (Dindo-Clavien ≥ 3) (Table 2).

## Discussion

Nutritional problems frequently exist in colorectal cancer patients, and this problem, which reaches the level of malnutrition in a significant part of them, adversely affects treatment and survival outcomes [1,5,6]. The importance of sarcopenia, one of the critical indicators of malnutrition, in patients undergoing colorectal surgery has attracted the attention of researchers, and in a recently published study evaluating 939 patients who underwent left colon and rectal surgery, 61% were found to have sarcopenia in the preoperative period [7]. Patients with sarcopenia necessitated more intraoperative transfusions, had lower albumin levels, and had prolonged hospital stays. Surgical site infections and anastomotic leakages were also significantly high in patients with sarcopenia. In addition, sarcopenia was found to have a negative impact on all survival parameters and mortality rates. More importantly, the Cox regression analysis revealed that preoperative sarcopenia was an independent predictive factor for worse OS [7].

In our study, in which we evaluated 38 patients who were operated on for left colon and rectal cancer, sarcopenia was present in 12 patients (31.5%) during the preoperative assessment. Comparable rates were found in previous studies evaluating the sarcopenia status of colorectal cancer patients before systemic treatment and surgery, and this rate even approached 40% in some series [8].

One of the essential inferences of our study is that a significant number of the patients were diagnosed at a locally advanced stage. At the time of diagnosis, 39.5% of our patients were at stage 3, and this finding showed us that we still have room for improvement in colorectal cancer screening at both the hospital and national levels. In addition, the American Cancer Society reported that the number of colorectal cancer patients diagnosed under the age of 55 has almost doubled in the last ten years [9].

Significant progress has been achieved in minimally invasive colorectal surgery since the 1990s, and we all comprehend that the advantages of laparoscopic surgery can be utilized without compromising oncological results [10]. The growing experience in robot-assisted surgeries inherently expands the indications, and robot-assisted surgery has become a good option for treating colorectal cancer patients. Published trials and meta-analyses also support the idea that robot-assisted surgery is a good, if not the best, option [11]. In our hospital, we prefer robot-assisted surgery in this patient population, and 34 patients (89.5%) in our cohort underwent robot-assisted surgery.

Seventeen of our patients (44.7%) had minor or major complications. Eight of these complications were found to be moderate to severe, and one anastomotic leak was observed (2.6%). Our complication rate seems relatively high compared to the published series. However, one of the reasons affecting this rate is the meticulousness of the postoperative follow-up and surveillance performed in our hospital. Our patient safety system aims to produce high-quality data and tries to record even the most minor problems. Although the overall rate seems high, only one anastomotic leak in our cohort in a male patient pleased us. Indeed, the male sex was reported to significantly increase anastomotic leak after minimally invasive rectal surgery [12], and in another study examining patients with anastomotic leakage, male gender and advanced age also increased the risk of leak and mortality [13]. Preoperative nutritional problems, fluid electrolyte imbalance, and low serum albumin levels were also correlated with an increased risk of anastomotic leak [13]. It was also reported that patients receiving neoadjuvant chemoradiotherapy also pose a risk of anastomotic leakage. The rate of anastomotic leakage varied greatly in published series, reported between 2% and 19%, but in reference centers and with experienced teams, it is 2-7% [14]. Although we have a robust anastomotic leakage rate, our study revealed that we should strive to reduce our overall complication rate after colorectal cancer surgery.

We aimed to evaluate the effect of sarcopenia on variables and complications by assigning the patients into two groups: those with and without preoperative sarcopenia. We revealed that there were more male patients in the group with preoperative sarcopenia, and the median age was higher in this group when compared with the non-sarcopenia group.

As discussed above, a higher rate of complications, especially anastomotic leakage, is seen in male and elderly patients [12,13,15]. We also know that mortality rates are higher in frail patient groups [15]. As a result of our study, we obtained clues that the possibility of preoperative sarcopenia is high in male and elderly patients, who already have a higher risk of complications and mortality; thus, monitoring those patients more closely and acting proactively in their treatment will be useful.

No difference was observed when the two groups were compared in terms of tumor localization, clinical stage, preoperative BMI values, and laboratory tests, including albumin. According to the methodology, the preoperative PMI values of both groups were significantly different. There was no difference between the two groups regarding the number of patients receiving neoadjuvant therapy or the pretreatment and preoperative BMI values of the patients in the subgroup of patients receiving neoadjuvant therapy. Although there was a decrease in BMI values in both groups during this treatment period, there was no difference when the groups were compared in terms of this change. It was observed that both groups were similar regarding the preferred surgical technique and postoperative pathological stages.

Localization of the tumors in the middle and distal rectum appears more common in patients without preoperative sarcopenia (11 out of 26 patients, 42%). In the non-sarcopenia group, this number is seen to be 4 out of 12 patients (33%). Knowing that surgeries on tumors located in the middle and distal rectum are more prone to complications and that there is a higher rate of neoadjuvant chemoradiotherapy use in these patients may explain the higher rate of "moderate to severe complications" in the non-sarcopenia group. On the other hand, when the overall complications were evaluated, the group with preoperative sarcopenia had a significantly higher rate when compared with the group without sarcopenia (58.8% vs. 38.4%, p=0.016). This finding indicated that monitoring patients with sarcopenia more closely during the perioperative period is essential. Early recognition of complications and prompt initiation of therapeutic interventions will improve our results in these high-risk patients. Contrary to our foresight, we revealed higher "moderate to severe complications" in patients who did not have preoperative sarcopenia. This finding must be validated with prospective, randomized, multicenter studies with more patients.

Our study has certain limitations. First, it is a retrospective, single-center, and non-randomized study with limited patients. We also do not have long-term follow-up data to conclude the impact of sarcopenia on the recurrence rate, disease, and overall survival.

## Conclusions

Our study revealed that sarcopenia was present in a significant number of patients who were operated on for left colon and rectal cancer. The rate was higher in males and elderly patients, and the risk of overall postoperative complications increased significantly in patients with preoperative sarcopenia. In consequence, the results of our study provide evidence that preoperative sarcopenia status is an important parameter to determine the patient's risk status, and patients with preoperative sarcopenia should be monitored more closely. Thus, we may be able to diagnose and intervene early in the complications.
